# Evaluation of antioxidant and anticancer properties of the seed extracts of *Syzygium fruticosum* Roxb. growing in Rajshahi, Bangladesh

**DOI:** 10.1186/1472-6882-13-142

**Published:** 2013-06-22

**Authors:** Shafiqul Islam, Samima Nasrin, Muhammad Ali Khan, ASM Sakhawat Hossain, Farhadul Islam, Proma Khandokhar, M Nurul Haque Mollah, Mamunur Rashid, Golam Sadik, Md Aziz Abdur Rahman, AHM Khurshid Alam

**Affiliations:** 1 Department of Pharmacy, University of Rajshahi, Rajshahi, 6205, Bangladesh; 2 Department of Pharmacy, Bangabandhu Sheikh Mujibur Rahman Science and Technology University, Gopalgonj, 8100, Bangladesh; 3 Department of Biochemistry and Molecular Biology, University of Rajshahi, Rajshahi, 6205, Bangladesh; 4 Department of Pharmaceutical and Biological Sciences, UCL School of Pharmacy, London, UK; 5 Department of Statistics, University of Rajshahi, Rajshahi, 6205, Bangladesh

**Keywords:** *Syzygium fruticosum* Roxb, Myrtaceae, Free radicals, Polyphenolics, Antioxidant activity, Anticancer activity

## Abstract

**Background:**

The use of plants and their derived substances increases day by day for the discovery of therapeutic agents owing to their versatile applications. Current research is directed towards finding naturally-occurring antioxidants having anticancer properties from plant origin since oxidants play a crucial role in developing various human diseases. The present study was designed to investigate the antioxidant and anticancer properties of *Sygygium fruticosum* (Roxb.) (abbreviated as SF).

**Methods:**

The dried coarse powder of seeds of SF was exhaustively extracted with methanol and the resulting crude methanolic extract (CME) was successively fractionated with petroleum ether, chloroform and ethyl acetate to get petroleum ether (PEF), chloroform (CHF), ethyl acetate (EAF) and lastly aqueous (AQF) fraction. The antioxidant activities were determined by several assays: total antioxidant capacity assay, DPPH free radical scavenging assay, hydroxyl radical scavenging assay, ferrous reducing antioxidant capacity and lipid peroxidation inhibition assay. The *in vivo* anticancer activity of SF was determined on Ehrlich’s Ascite cell (EAC) induced Swiss albino mice.

**Results:**

All the extractives showed strong antioxidant activities related to the standard. The total antioxidant capacity (TAC) of the fractions was in the following order: EAF>AQF>CME>PEF>CHF. The TAC of EAF at 320 μg/mL was 2.60±0.005 which was significantly higher (*p* < 0.01) than that of standard catechin (1.37 ± 0.005). The ferrous reducing antioxidant capacity of the extracts was in the following order: EAF>AQF>CME>AA>CHF>PEF. In DPPH free radical scavenging assay, the IC_50_ value of EAF was 4.85 μg/mL, whereas that of BHT was 9.85 μg/mL. In hydroxyl radical scavenging assay and lipid peroxidation inhibition assay, the EAF showed the most potent inhibitory activity with IC_50_ of 43.3 and 68.11 μg/mL, respectively. The lipid peroxidation inhibition assay was positively correlated (*p* < 0 .001) with both DPPH free radical scavenging and hydroxyl radical scavenging assay. The total phenolic contents of SF were also positively correlated (*p* < 0 .001) with DPPH free radical scavenging, hydroxyl radical scavenging and lipid peroxidation inhibition assay. Based on antioxidant activity, EAF was selected for cytotoxic assay and it was found that EAF inhibited 67.36% (*p* < 0.01) cell growth at a dose of 50 mg/kg (ip) on day six of EAC cell incubation.

**Conclusions:**

Our results suggest that EAF of seeds of SF possess significant antioxidant and moderate anticancer properties. Seeds of SF may therefore be a good source for natural antioxidants and a possible pharmaceutical supplement.

## Background

It is increasingly being realized that many of today’s diseases are due to the oxidative stress (OS) that results from an imbalance between formation and neutralization of prooxidants. OS is initiated by free radicals like hydroxyl, peroxyl and superoxide radicals, which become stable through electron pairing with biological macromolecules such as proteins, lipids and DNA in healthy human cells and cause protein and DNA damage along with lipid peroxidation. The damage caused by OS has been implicated as a potential contributor to the pathogenesis of cancer, diabetes, atherosclerosis, cardiovascular diseases, ageing and inflammatory diseases [1,2]. The damage can become more widespread due to weakened cellular antioxidant defense systems. All biological systems have antioxidant defense mechanism that protects against oxidative damages and repairs enzymes to remove damaged molecules. However, this natural antioxidant defense mechanism can be inefficient; hence dietary intake of antioxidant is important.

Antioxidants are substances that prevent damage to cells caused by free radicals by supplying electron to these free radicals. This stabilizes the molecule, thus preventing damage to other cells. Antioxidants also turn free radicals into waste by products, and they eventually get eliminated from the body. However, consumption of fruits and vegetables is known to lower the risk of several diseases, such as cancer, cardiovascular diseases and stroke caused by OS [3], and such health benefits are mainly imposed due to the presence of phytochemicals, such as polyphenols, carotenoids and vitamin E and C [4].

Although the phenolic compounds are commonly found in both edible and non edible herbs, cereals, fruits, vegetables, oils, spices and other plant materials [5,6], scientific information on antioxidant properties of endemic plants, limited to certain regions and known only by local populations, is still rather scarce. Therefore, the assessment of such properties remains an interesting and useful task, particularly to find new promising sources of natural antioxidants for functional foods and/or nutraceuticals [6,7].

*Syzygium fruticosum* (family Myrtaceae) is one of 1100 species in the genus *Syzygium* and is widely spread in India, Myanmar, China, Thailand and Bangladesh [8]. It is used in some countries as folk remedy for the treatment of diabetes, stomachic, and bronchitis [9,10]. To our knowledge from literatures there is no work about phytochemical contents and biological activities of *Syzygium fruticosum*. However, literature review on *Syzygium* genus showed that the various species of *Syzygium* possess a variety of biological activities. The leaves of *Syzygium cumini* showed antioxidant, anti-allergic, anti-inflammatory and analgesic propertie [9,10]. The seed extracts of *S*. *aromaticum*, *S*. *jambos* and *S*. *aqueum* and the fruit and bark extracts of *S*. *aromaticum* showed antidiabetic, antihyperlipidemic, gastroprotective, antioxidant, anti-allergic, and analgesic activities [10-14].

To reveal the potential of medicinal plants available in Bangladesh, as a part of our ongoing research [15], we selected *Syzygium fruticosum* as the information of medicinal values of *S*. *fruticosum* is still lacking in the literature. Therefore, this study was carried out to enrich the information of the medicinal property of *S*. *fruticosum* in terms of antioxidant and anticancer as well as its polyphenoic contents in the literature.

## Methods

### Collection of plant materials

Seeds of SF were collected from Rajshahi University Campus, Rajshahi, Bangladesh in May, 2011 and were identified by an expert taxonomist at National Herbarium, Dhaka, Bangladesh where a voucher specimen was deposited (Accession number: 1326). Plant materials were then washed with fresh water to remove dirty materials and were shade dried for several days with occasional sun drying. The dried materials were ground into coarse powder by grinding machine, and the materials were stored at room temperature for future use.

### Preparation of extract

About 500 gm of dried powdered plant materials were taken in an amber colored extraction bottle (2.5 liter capacity) and the materials were soaked with methanol (1L × 3 times). The sealed bottle was kept for 7 days with occasional shaking and stirring. The combined extracts were filtered through cotton and then Whatman No.1 filter papers and were concentrated with a rotary evaporator under reduced pressure at 45°C to afford 40 gm crude seed extract. The extract was then fractionated by pet-ether, chloroform, ethyl acetate and finally with water to obtain petroleum ether fraction (PEF, 13.54 gm), chloroform fraction (CHF, 8.28 gm), ethyl acetate fraction (EAF, 7.68 gm) and aqueous fraction (AQF, 10.50 gm).

### Chemicals

1,1-diphenyl-2-picrylhydrazyl (DPPH), potassium ferricyanide, potassium acetate, phosphate buffer, catechin (CA), ferrous ammonium sulphate, butylated hydroxytoluene (BHT), gallic acid (GA), ascorbic acid (AA), AlCl_3_, Trichloro acetic acid (TCA), sodium phosphate, ammonium molybdate, tannic acid, quercetin (QU), DMSO, EDTA, thiobarbituric acid (TBA), acetyl acetone and FeCl_3_ were purchased from Sigma Chemical Co. (St. Louis, MO, USA); vanillin was obtained from BDH; Folin-Ciocalteus’s phenol reagent and sodium carbonate were obtained from Merck (Damstadt, Germany).

### Estimation of total phenolics

Total phenolic contents of the extracts were determined by the modified Folin-Ciocalteu method described by Wolfe et al. [16]. An aliquot of the extracts/standard was mixed with 2 ml Folin-Ciocalteu reagent (previously diluted with water 1:10 v/v) and 2 ml (75 g/l) of sodium carbonate. The tubes were vortexed for 15 seconds and allowed to stand for 20 minutes at 25°C for color development. Absorbance was then measured at 760 nm UV-spectrophotometer (Shimadzu, USA). Samples of extracts/standard were evaluated at a final concentration of 0.1 mg/mL. Total phenolic contents were expressed in terms of gallic acid equivalent, GAE (standard curve equation: y = 0.011x+0.066, R^2^ = 0.998), mg of GA/g of dry extract.

### Determination of total flavonoids

Total flavonoids were estimated using the method described by Ordonez et al. [17]. To 0.5 ml of samples/standard, 1.5 ml of methanol, 100 μl of 10% aluminum chloride, 100 μl of 1M potassium acetate solution and 2.8 ml of distilled water was added. After one hour 30 minutes of incubation at room temperature (RT), the absorbance was measured at 420 nm. The samples/standard was evaluated at a final concentration of 0.1 mg/mL. Total flavonoid contents were expressed in terms of catechin equivalent, CAE (standard curve equation: y = 0.003x+0.022, R^2^ = 0.997), mg of CA/g of dry extract.

### Determination of total flavonols

Total flavonols in the plant extracts were estimated using the method of Kumaran and Karunakaran [18]. To 2.0 ml of sample/standard, 2.0 ml of 2% AlCl_3_ ethanol and 3.0 ml (50 g/L) sodium acetate solutions were added. The absorption at 440 nm was read after 2.5 hours at 20°C. Extractives/standard were evaluated at a final concentration of 0.1 mg/mL. Total content of flavonols was expressed in terms of quercetin equivalent, QUE (standard curve equation: y = 0.0255x+0.0069, R^2^ = 0.9995), mg of QU/g of dry extract.

### Determination of total proanthocyanidins

Determination of content of proanthocyanidins was based on the procedure reported by Sun et al. [19]. A volume of 0.5 ml of 0.1 mg/mL of extracts/standard solution was mixed with 3 ml of 4% vanillin-methanol solution and 1.5 ml hydrochloric acid; the mixture was allowed to stand for 15 minutes. The absorbance was measured at 500 nm. Samples/standard was evaluated at a final concentration of 0.1 mg/mL. Total content of proanthocyanidins was expressed in terms of catechin equivalent, CAE (standard curve equation: y = 0.567x-0.024, R^2^ = 0.9801), mg of CA/g of dry extract.

### Determination of antioxidant activity

#### Determination of total antioxidant capacity

Total antioxidant capacity (TAC) of samples/standard was determined by the method reported by Prieto et al. [20] with some modifications. 0.5 ml of samples/standard at different concentrations was mixed with 3 ml of reaction mixture containing 0.6 M sulphuric acid, 28 mM sodium phosphate and 1% ammonium molybdate into the test tubes. The test tubes were incubated at 95°C for 10 minutes to complete the reaction. The absorbance was measured at 695 nm using a spectrophotometer against blank after cooling at RT. CA was used as standard. A typical blank solution contained 3 ml of reaction mixture and the appropriate volume of the same solvent used for the samples/standard were incubated at 95°C for 10 minutes and the absorbance was measured at 695 nm. Increased absorbance of the reaction mixture indicated increase total antioxidant capacity.

### *Determination of ferrous reducing antioxidant capacity*

The ferrous reducing antioxidant capacity of samples/standard was evaluated by the method of Oyaizu [21]. 0.25 ml samples/standard solution at different concentrations, 0.625 ml of potassium buffer (0.2 M) and 0.625 ml of 1% potassium ferricyanide [K_3_Fe (CN)_6_] solution were added into the test tubes. The reaction mixture was incubated for 20 minutes at 50°C to complete the reaction. Then 0.625 ml of 10% TCA solution was added into the test tubes. The total mixture was centrifuged at 3000 rpm for 10 minutes. After which, 1.8 ml supernatant was withdrawn from the test tubes and was mixed with 1.8 ml of distilled water and 0.36 ml of 0.1% FeCl_3_ solution. The absorbance of the solution was measured at 700 nm using a spectrophotometer against blank. A typical blank solution contained the same solution mixture without plant extracts/standard was also incubated under the same condition, and the absorbance of the blank solution was measured at 700 nm. Increased absorbance of the reaction mixture indicated increase reducing capacity.

#### DPPH free radical scavenging assay

Free radical scavenging activity was determined by DPPH radical scavenging assay as described by Choi et al. [22]. A solution of 0.1 mM DPPH in methanol was prepared and 2.4 ml of this solution was mixed with 1.6 ml of extractives in methanol at different concentrations. The reaction mixture was vortexed thoroughly and left in the dark at RT for 30 minutes. The absorbance of the mixture was measured spectrophotometrically at 517 nm. BHT was used as reference standard. Percentage DPPH radical scavenging activity was calculated by the following equation:

$$\begin{array}{l} \left(\%\;\mathrm{DPPH}\;\mathrm{radical}\;\mathrm{scavenging}\;\mathrm{activity}\right)\\ \;=\left\{\left(A_{o}{–A}_{1}\right)/A_{o}\right\}\times 100 \end{array}$$

where A_0_ is the absorbance of the control, and A_1_ is the absorbance of the extractives/standard. Then % of inhibition was plotted against concentration, and from the graph IC_50_ was calculated.

#### Hydroxyl radical scavenging activity

Hydroxyl radical scavenging activity of the extractives/standard was determined by the method of Klein et al. [23] with a slight modification. 0.5 ml of extractives/standard at different concentrations was taken in different test tubes. 1 ml of Fe-EDTA solution (0.13% ferrous ammonium sulphate and 0.26% EDTA), 0.5 ml of 0.018% EDTA solution, 1 ml of 0.85% DMSO solution and 0.5 ml of 22% AA were added into each of the test tubes. The test tubes were capped tightly and warm at 85°C for 15 minutes into the water bath. After incubation, the test tubes were uncapped and 0.5 ml ice cold TCA (17.5%) was added to each of test tubes immediately. Three ml of nash reagent (7.5 gm of ammonium acetate, 300 μl glacial acetic acid and 200 μl acetyl acetone were mixed and made up to 100 ml) was added into all of the test tubes and incubated at RT for 15 minutes. Absorbance was taken at 412 nm wave length by UV-spectrophotometer. Percentage hydroxyl radical scavenging activity was calculated by the following equation:

$$\begin{array}{l} \%\;\mathrm{hydroxyl}\;\mathrm{radical}\;\mathrm{scavenging}\;\mathrm{activity}\\ \;=\left\{\left(A_{o}{–A}_{1}\right)/A_{o}\right\}\times 100 \end{array}$$

where A_0_ is the absorbance of the control, and A_1_ is the absorbance of the extractives/standard. Then % of inhibition was plotted against concentration, and from the graph IC_50_ was calculated.

#### Lipid peroxidation inhibition assay

The lipid peroxidation inhibition assay was determined according to the method described by Liu et al. [24] with a slight modification. Excised rat liver was homogenized in buffer and then centrifuged to obtain liposome. 0.5 ml of supernatant, 100 μl 10 mM FeSO_4_, 100 μl 0.1 mM AA and 0.3 ml of extractives/standard at different concentrations were mixed to make the final volume 1 ml. The reaction mixture was incubated at 37°C for 20 minutes. One ml of (28%) TCA and 1.5 ml of (1%) TBA was added immediately after heating. Finally, the reaction mixture was again heated at 100°C for 15 minutes and cooled at RT. After cooling, the absorbance was taken at 532 nm. Percentage inhibition of lipid peroxidation was calculated by the following equation:

$$\begin{array}{l} \%\;\mathrm{lipid}\;\mathrm{peroxidation}\;\mathrm{inhibition}\\ \;=\left\{\left(A_{o}{–A}_{1}\right)/A_{o}\right\}\times 100 \end{array}$$

where A_0_ is the absorbance of the control, and A_1_ is the absorbance of the extractives/standard.

Then % of inhibition was plotted against concentration, and IC_50_ was calculated from the graph.

### Determination of anticancer activity

#### Cell growth inhibition

*In vivo* tumor cell growth inhibition was carried out by the method previously described by Sur et al. [25]. Protocol used in this study for the use of mice as animal model for cancer research was approved by the Institutional Animal, Medical Ethics, Biosafety and Biosecurity Committee (IAMEBBC) for Experimentations on Animal, Human, Microbes and Living Natural Sources (225/320-IAMEBBC/IBSc), Institute of Biological Sciences, University of Rajshahi, Bangladesh.

For this study, 4 groups of mice (6 in each group) were used. For therapeutic evaluation, 14 × 10^5^ Ehrlich’s Ascites cells (EAC)/mouse was inoculated into each group of mice on the first day. Treatment was started after 24 hours of EAC inoculation and continued for 5 days. Group 1 to 2 received the test compound at doses 25 mg/kg and 50 mg/kg, respectively per day per mouse. In each case, the volume of the test solutions injected intraperitoneally (i.p.) was 0.1ml/day per mouse. Group 3 received standard bleomycin (0.3 mg/kg, i.p) and were considered as positive control. Finally the group 4 mice were treated with the vehicle (normal saline) and were considered as untreated control. The mice were sacrificed on the 6^th^ day after transplantation and tumor cells were collected by repeated i.p. wash with 0.9% saline. Viable tumor cells per mouse of the treated group were compared with those of control.

The cell growth inhibition was calculated using the following formula:

$$\%\;\mathrm{Cell}\;\mathrm{growth}\;\mathrm{inhibition}=\left(1–\frac{\mathrm{Tw}}{\mathrm{Cw}}\right)\times 100$$

where, Tw = Mean of number of tumor cells of the treated group of mice and Cw = Mean of number of tumor cells of the control group of mice.

### Statistical analysis

All analyses were carried out in triplicates. Data were presented as mean ± SD. To evaluate significant relationships between experimental parameters by correlation and regression analysis, the *F*- and *t*-*tests* (*p* < 0.001) were used. Free R-software version 2.15.1 (http://www.r-project.org/) and Microsoft Excel 2007 (Roselle, IL, USA) were used for the statistical and graphical evaluations.

## Results

### Determination of TAC and ferrous reducing antioxidant capacity

**Figure 1:**
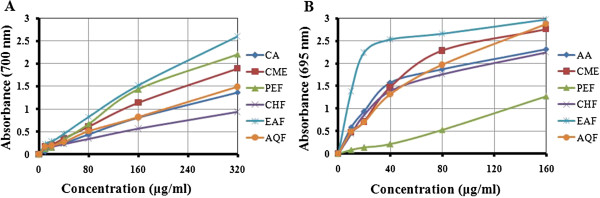
**Determination of (A) total antioxidant capacity and (B) ferrous reducing antioxidant capacity of CME and its various fractions (PEF, CHF, EAF and AQF).** Data expressed as mean ± SD (*n* = 3, *p* < .05) for all tested dosages.

The TAC of CME and its four fractions of seeds of SF were shown in Figure 1A. CME of seeds of SF showed higher antioxidant activity compared to reference standard CA at all the concentrations. The absorbance of CME, PEF, CHF, EAF, AQF, and standard CA were 1.90, 2.21, 0.96, 2.60, 1.49 and 1.37, respectively at 320 μg/ml. The TAC of EAF was significantly higher (*p* < 0.01) than standard CA. The extractives were found to increase the total antioxidant activity with the increasing concentration of the extracts.

The Ferrous reducing antioxidant capacity of CME and it’s four fractions are shown in Figure 1B. At 160 μg/ml, the absorbance of CME, PEF, CHF, EAF, AQF, and standard AA were 2.77, 1.28, 2.24, 2.98, 2.89 and 2.32, respectively. A higher absorbance indicates a higher reducing power, hence CME, EAF and AQF showed higher reducing activity than standard AA. The ferrous reducing capacity of EAF was significantly higher (*p* < 0.01) than standard AA. On the other hand, PEF and CHF had mild to moderate iron reducing capacity. The reducing activity increased with the increasing concentration of the extracts.

### DPPH radical scavenging activity

**Figure 2:**
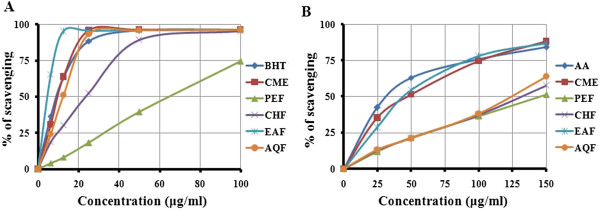
**Determination of (A) DPPH radical scavenging activity and (B) OH radical scavenging activity of CME and its various fractions (PEF, CHF, EAF and AQF).** Data expressed as mean ± SD (*n* = 3, *p* < .05) for all tested dosages.

Figure 2A showed free radical scavenging activity of the CME and its four fractions. At a concentration of 25 μg /ml, the scavenging activity of the CME, EAF and AQF were 95.95, 95.68 and 93.44%, respectively, while at the same concentration, the activity of BHT was 88.51%. Thus, CME, EAF and AQF exhibited significant free radical scavenging activity (Figure 2A). The scavenging activity of the PEF and CHF was in moderate level when compared with BHT (Figure 2A). The IC_50_ of CME, PEF, CHF, EAF and AQF were 9.90, 63.0, 23.7, 4.85 and 10.0 μg/ml, respectively. The IC_50_ of BHT was 9.85 μg/ml, which was almost double than the IC_50_ of EAF (4.85 μg/ml). The inhibitory activity of different extractives and BHT were in the following order: EAF > BHT > CME> AQF > CHF > PEF. Our results revealed that the EAF had higher scavenging activity than that of other extractives, even higher than BHT and CME and AQF had similar activity with BHT.

### Hydroxyl radical scavenging activity

At a concentration of 150 μg /ml, the scavenging activity of CME and its four fractions PEF, CHF, EAF and AQF reached 88.27, 51.00, 57.83, 87.13 and 63.90%, respectively; while at the same concentration, the activity of AA was 84.24% (Figure 2B). The IC_50_ of CME, PEF, CHF, EAF, AQF and AA were 50.23, 145.27, 133.43, 43.31, 124.61 and 32.11 μg/ml, respectively. The result demonstrates that CME and EAF significantly scavenged hydroxyl radicals when compared with standard AA.

### Lioid peroxidation inhibition assay

**Figure 3:**
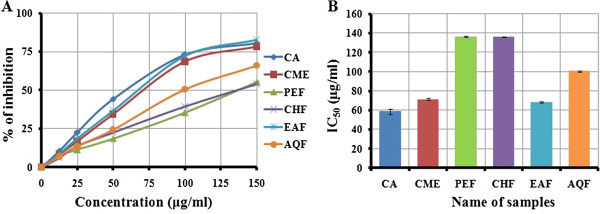
**Determination of (A) lipid peroxidation inhibition of CME and its various fractions (PEF, CHF, EAF and AQF) and (B) IC**_**50 **_**of CME and its various fractions (PEF, CHF, EAF and AQF) determined from lipid peroxidation inhibition assay.** Data expressed as mean ± SD (*n* = 3, *p* < .05) for all tested dosages.

**Figure 4:**
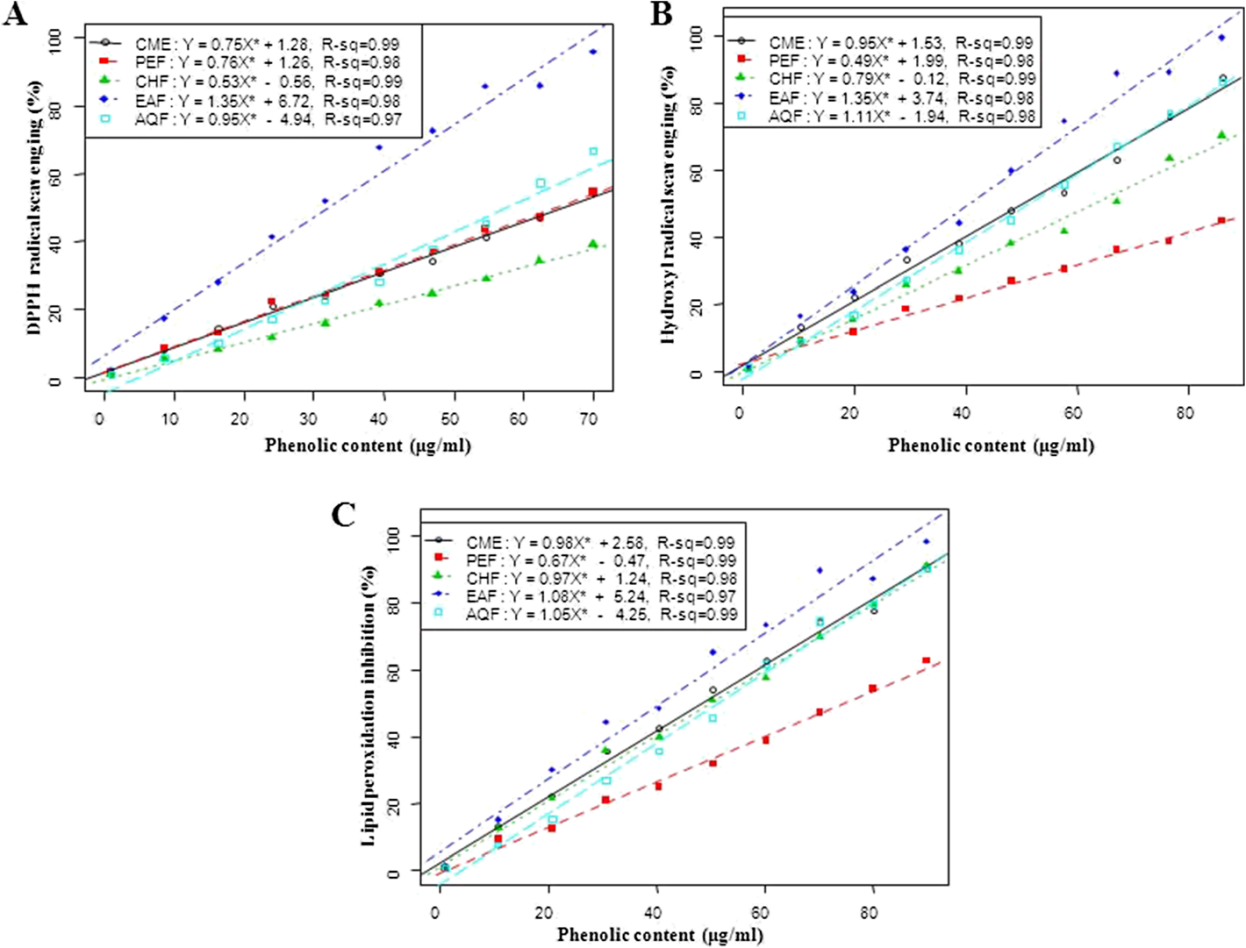
**Relationship of total phenolic contents with (A) % DPPH free radical scavenging, (B) % hydroxyl radical scavenging activity and (C) % lipid peroxidation inhibition.** Data expressed as mean ± SD (*n* = 3, *p* < .001).

The lipid peroxides scavenging activity of CME of seeds of SF was investigated and compared with standard CA. At a concentration of 150 μg/ml, the scavenging activity of CME and its fractions PEF, CHF, EAF and AQF were 78.20, 55.36, 53.99, 82.63 and 66.10%, respectively; whereas the activity of CA was 80.54% (Figure 3A). The EAF exhibited higher activity than other extractives, even though higher than standard CA. The IC_50_ of CME, PEF, CHF, EAF and AQF were 71.50, 136.34, 136.21, 68.11 and 100.31 μg/ml, respectively; on the other hand, the IC_50_ of CA was 58.5 (Figure 3B). Significant correlations (*p* <*0*.*001*) were observed between % lipid peroxidation inhibition and % hydroxyl radical scavenging (Figure 4).

### Total phenolic, flavonoids, flavonol and proanthocyanidin contents

**Figure 5:**
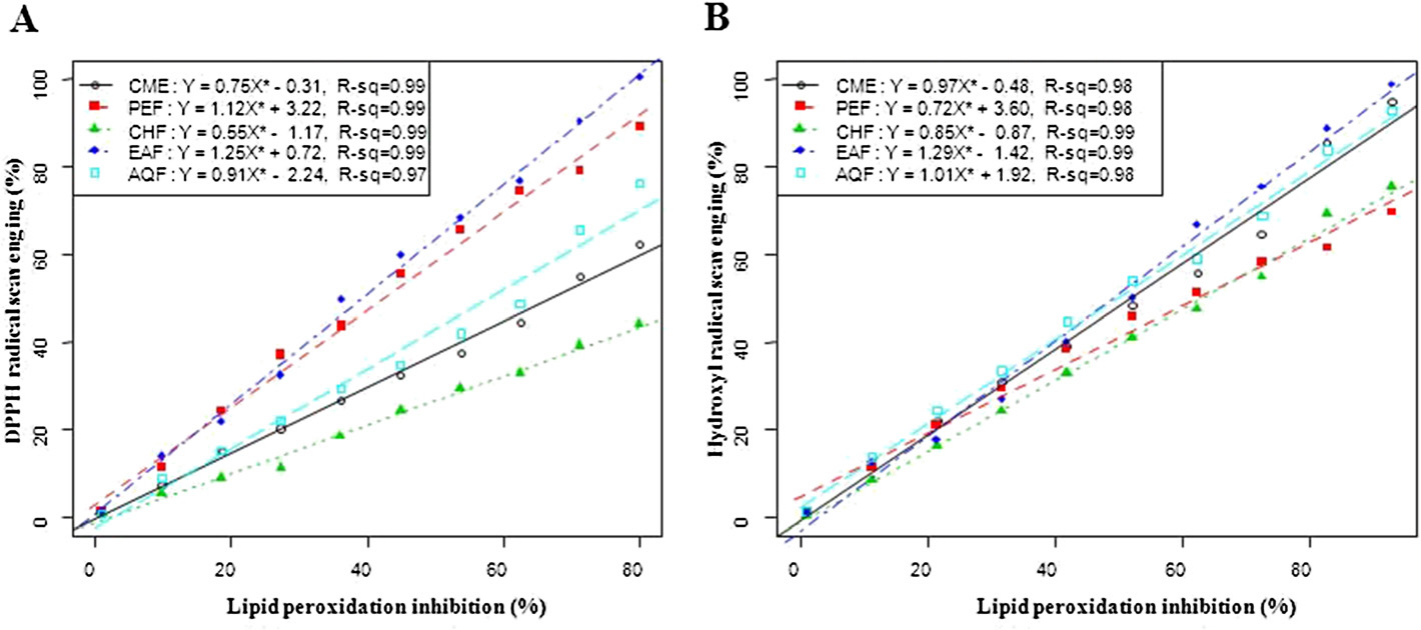
**Relationship of % lipid peroxidation inhibition with (A) % DPPH free radical scavenging and (B) % hydroxyl radical scavenging activity.** Data expressed as mean ± SD (*n* = 3, *p* < .001).

Table 1 shows the total polyphenols contents in the CME and its four fractions: PEF, CHF, EAF and AQF. Strong correlation (*p* <*0*.*001*) of total phenolic content of the extractives with free radical (^•^OH) scavenging efficiency and % lipid peroxidation inhibition were observed (Figure 5).

**Table 1:** **Polyphenol contents of CME and its various fractions: PEF**, **CHF**, **EAF and AQF**

**Polyphenols**	**CME**	**PEF**	**CHF**	**EAF**	**AQF**
Phenolics^*a*^	301.63 ± 4.36^1^	17.56 ± 1.41	118.13 ± 1.61	526.22 ± 1.55	266.88 ± 2.78
Flavonoids^*b*^	219.88 ± 15.17	152.13 ± 6.34	128.21 ± 9.38	612.75 ± 5.37	185.71 ± 11.35
Flavonols^*c*^	119.38 ± 1.24	185.48 ± 1.19	149.01 ± 2.78	220.38 ± 1.26	132.54 ± 1.77
Proanthocyanidins^*b*^	10.32 ± 1.31	12.36 ± 0.04	11.94 ± 0.25	44.68 ± 0.05	33.33 ± 0.07

NB: ^1^Each value is the average of three analyses ± standard deviation. *a*, *b* and *c* expressed in terms of GAE, CAE and QUE, respectively (mg of GA, CA and QU/g of dry extract, respectively).

### Tumor cell growth inhibition

Since EAF showed the strongest antioxidant activity in all antioxidant tests, it was chosen for *in vivo* tumor cell growth inhibition at various doses (25 mg/kg and 50.0 mg/kg, i.p). Maximum cell growth inhibition (67.36%, *p* < 0.01) was found after treatment with EAF at dose 50.0 mg/kg (i.p) on day six of tumor inoculation. On the other hand, standard bleomycin at dose 0.3 mg/kg i.p inhibited the tumor cell growth by 83.81% (Table 2). This result implies that the EAF had moderate anticancer activity, and the plant might therefore be considered as an effective source of active chemopreventive agents.

**Table 2:** **Effect of EAF on EAC cell growth inhibition in mice** (***in vivo***)

**Name of Exp.**	**Nature of the drug**	**Dose mg/kg/day (i.p)**	**No. of EAC cells in mouse on day 6 after tumour cell inoculation**	**% of cell growth inhibition**
Control (EAC cell bearing mice)	-	-	(3.83 ± 0.57) ×10^7^	-
Bleomycin	Standard	0.3 mg/kg	(0.62 ± 0.05) × 10^7**^	83.81
EAF	Experimental	25 mg/kg	(1.9 ± 0.21) × 10^7*^	50.39
		50 mg/kg	(1.25 ± 0.11) × 10^7**^	67.36

Number of mice in each case (n=6); the results were shown as mean ± SEM. Where significant values are *p < 0.05 and ^**^p < 0.01

## Discussion

### Total antioxidant capacity

The total antioxidant potentials of seeds of SF extracts were estimated from their ability to reduce the reduction of Mo (VI) to Mo (V) and subsequent formation of a green phosphate/Mo (V) complex at acidic pH. The reducing ability of the extractives was in the range of 0.94±0.002-2.60±0.005 μm green phosphate/Mo (V) (Figure 1A). All the fractions showed a good total antioxidant activity, which was concentration-dependent. The antioxidant activity of EAF was significantly higher (*p* < .05) than standard antioxidant. The antioxidant capacity may be attributed to their chemical composition and phenolic content. Jayaprakasha et al. [26] indicated that the total antioxidant activity of citrus was due to the presence of phenolics, flavonoids and ascorbic acid.

### Ferrous reducing antioxidant capacity

Reducing power is also widely used in evaluating antioxidant activity of plant polyphenols. The reducing power is generally associated with the presence of reductones, which exert antioxidant action by breaking the free radical chains by donating a hydrogen atom. In this assay, the presence of reductants in the antioxidant sample causes the reduction of the Fe^3+^/ferricyanide complex to the Fe^2+^/ferrous form, so the reducing power of the sample can be monitored by measuring the formation of Perl’s Prussian blue at 700 nm [27]. The reducing ability of the extractives was in the range of 1.47±0.004-2.98±0.002 μm Fe (II)/g. The EAF exhibited strong reducing power and was higher than other fractions, even significantly higher (*p* < .05) than AA as shown in Figure 1B. The reducing power of EAF is probably due to the presence of phenolic compounds which might act as electron donors.

### DPPH radical scavenging activity

The effect of antioxidants on DPPH radicals is thought to be due to their hydrogen donating ability [22]. Radical scavenging activities are very important to prevent the deleterious role of free radical in different diseases including cancer. DPPH free radical scavenging is an accepted mechanism by which antioxidants act to inhibit lipid peroxidation. This method has been used extensively to predict antioxidant activities because of the relatively short time required for analysis. The DPPH radical scavenging activity of all the fractions from SF seeds increased with increase in fraction concentration (Figure 2A). The IC_50_ of EAF was significantly higher (*p* < .01) than that of other fractions and BHT with the order of EAF > BHT > CME > AQF > CHF > PEF. It has been found that phenolics, flavonoids and tocopherols reduce the DPPH radicals by their hydrogen donating ability [28,29]. The results obtained in this investigation reveal that all the fractions from SF seeds are free radical scavengers and able to react with the DPPH radical, which might be attributed to their electron donating ability.

### Hydroxyl radical scavenging activity

The mutagenic capacity of free radicals is due to the direct interactions of hydroxyl radicals with DNA and therefore playing an important role in cancer formation [30]. Hydroxyl radicals can be generated by biochemical reaction. Superoxide radical is converted by superoxide dismutase to hydrogen peroxide, which can subsequently produce extremely reactive hydroxyl radicals in the presence of divalent metal ions, such as iron and copper. The results obtained in this study demonstrate that EAF of seeds of SF had appreciable hydroxyl radical scavenging activity when compared with standard antioxidant BHT (Figure 2B) and could be served as anticancer agent by inhibiting the interaction of hydroxyl radical with DNA. The ability of the extracts to quench hydroxyl radicals might directly be related to the prevention of lipid peroxidation.

### Lipid peroxidation inhibition assay

Reactive oxygene species induce membrane damage by peroxidising lipid moiety, specially the polyunsaturated fatty acids with a chain reaction known as lipid peroxidation [31]. The initial reaction generates a second radical, which in turn can react with a second macromolecule to continue the chain reaction leads to functional abnormalities of cells. Lipid peroxidation has been reported to be elevated in the cancer [32]. In this study, lipid peroxidation of mouse liver homogenates was induced by ferric ion plus ascorbic acid. The CME of seeds of SF and its four fractions especially EAF had appreciable lipid peroxidation inhibition activity (Figure 3). The SF extracts can prevent the cell abnormalities caused by cancer through breaking down of chain reactions responsible for lipid peroxidation. The lipid peroxidation inhibition by the EAF fraction was significantly correlated with hydroxyl radical scavenging (Figure 4). This result reveals that the extractives differentially inhibit lipid peroxidation by virtue of their varying degrees of free radical quenching potential. Thus, SF is a good source for antioxidant thereby can be used as anticancer agent.

### Effect of antioxidant on EAC-induced tumor cells

Antioxidants neutralize free radicals, which is a natural by-product of normal cell processes. Free radicals are molecules with incomplete electron shells which make them more chemically reactive than those with complete electron shells. In humans, the most common form of free radicals is oxygen. When an oxygen molecule (O_2_) becomes electrically charged or radicalized, it tries to steal electrons from other molecules, causing damage to the DNA and other molecules. Over time, such damage may become irreversible and lead to disease including cancer. Antioxidants are often described as “mopping up” free radicals, meaning they neutralize the electrical charge and prevent the free radical from taking electrons from other molecules thereby prevent cancer. Several laboratory evidences from chemical, cell culture and animal studies indicate that antioxidants may slow or possibly prevent the development of cancer [33]. In this study, the anticancer activity of EAF at low dose (below 25 mg/kg) on EAC-induced cancer in mice was not observed (data not shown). Although the EAF inhibited the growth of cancer cells only at higher concentrations (25 and 50 mg/kg), it exhibited comparable anticancer activity with bleomycin (Table 2), hence EAF might be a good source for isolating anticancer agent.

### Total phenolic, flavonoids, flavonols and proanthocyanidin contents

Polyphenols are the most abundant antioxidants in the plant kingdom, and they have been claimed to have anti cancer property [34]. The antioxidant activity of the polyphenolic compounds is believed to be mainly due to their redox properties [35], which play an important role in adsorbing and neutralizing free radicals, quenching singlet and triplet oxygen or decomposing peroxides. Flavonoids are the most ubiquitious groups of plant secondary metabolites and have good antioxidant potential. Flavonoids have been shown to possess antimutagenic and antimalignant effect [36]. Furthermore, flavonoids have a chemopreventive role in cancer through their effect on signal transduction in cell proliferation and angiogenesis [37]. Dietary flavonols and proanthocyanidins in particular offer significant cardiovascular health benefits [38]. Proanthocyanidin-rich extract has preventive actions on diseases, such as atherosclerosis, gastric ulcer, large bowel cancer, cataracts and diabetes [39]. Results obtained in the present study revealed that the level of these phenolic compounds in seeds of SF were significant (Table 1). Our findings strongly suggest that the phenolics are important components of this plant, and some of its pharmacological effects like anticancer activity could be attributed to the presence of these valuable constituents.

## Conclusions

The present study indicated that the ethyl acetate fraction of *Syzygium fruticosum* possessed the highest phenolic content than other fractions. Also, the EAF exhibited strong antioxidant and moderate anticancer activities, which were comparable to the commercial antioxidants BHT, CA and AA and the anticancer drug bleomycin. This seems that the *Syzygium fruticosum* extract can be used as natural antioxidant and anticancer agent. Further investigation is being carried out to identify and characterize the inherent phenolic compounds responsible for the antioxidant and anticancer activities from the ethyl acetate fraction of *Syzygium fruticosum*.

## Abbreviations

AA: Ascorbic acid; AQF: Aqueous fraction; CAE: Catechin equivalent; CHF: Chloroform fraction; CME: Crude methanolic extract; DPPH: 1,1-diphenyl-2-picrylhydrazine; EAC: Ehrlich’s Ascites cells; EAF: Ethyl acetate fraction; GA: Gallic acid; GAE: Gallic acid equivalent; OS: Oxidative stress; PEF: Petroleum ether fraction; QE: Quercetin equivalent; ROS: Reactive oxygen species; RT: Room temperature; SF: *Syzygium fruticosum*; TAC: Total antioxidant capacity; TCA: Trichloro acetic acid.

## Competing interests

The authors declare that they have no competing interests.

## Authors’ contributions

SI and SN, Designed the study and carried out the tests under the supervision of AHMKA. MAK, Carried out the lipid peroxidation inhibition assay. ASMSH, Helped to carry out the assay. FI, Carried out the anticancer assay. PK, Checked the grammatical errors and corrected the final manuscript. NHM, Performed statistical (correlation and regression) analysis. MR, Helped to carry out the assay. GS, Helped to coordinate the biological assay and draft the manuscript. AAR, Helped to carry out the extraction process and prepare the manuscript. All authors read and approved the final manuscript.

## Pre-publication history

The pre-publication history for this paper can be accessed here:

http://www.biomedcentral.com/1472-6882/13/142/prepub
